# Factors associated with Chinese herbal medicine use among middle-aged and older women with arthritis: evidence from China

**DOI:** 10.1038/s41598-022-16927-4

**Published:** 2022-07-22

**Authors:** Lu Yang, David Sibbritt

**Affiliations:** 1 Faculty of Sociology and Population Studies, Nanjing University of Posts and Telecommunications, 9 Wenyua Road, Qixia District, Nanjing, 210023 China; 2 School of Public Health, Faculty of Health, University of Technology Sydney, Level 8, Building 10, 235-253 Jones St, Sydney, NSW 2007 Australia

**Keywords:** Rheumatic diseases, Health services

## Abstract

Chinese herbal medicine (CHM) has been used for arthritis in China and elsewhere across the world. However, knowledge about the prevalence and profile of middle-aged and older women who used CHM for arthritis in China is limited. This study aims to identify potentially important insights into the factors associated with CHM use amongst middle-aged and older women with arthritis in China. Data were drawn from the China Health and Retirement Longitudinal Study (CHARLS), a population-based survey of Chinese adults aged 45 years or older, comprising 10,833 Chinese women who completed a questionnaire in 2015. Stepwise multiple logistic regression modeling was conducted to determine the key factors (demographic, health condition, and health services use) predicting the use of CHM for the treatment of arthritis. Results revealed that 17.2% of women with arthritis were taking CHM for their arthritic symptoms. Women with arthritis who used CHM were more likely to experience finger pain (OR = 1.70), had difficulty in stooping, kneeling, crouching (OR = 1.40), visited a Traditional Chinese hospital (OR = 2.22), consulted massage therapists (OR = 2.06) and/or had experienced a fall (OR = 1.41). The prevalence of CHM use is high amongst middle-aged and older Chinese women with arthritis. Given the high risk of functional disability and impaired mental health, further research is needed to explore the potential health benefits of CHM for women with arthritis in order to help facilitate the efficacious and safe use of CHM alongside conventional medical care.

## Introduction

Arthritis is one of the leading causes of pain, disability, and health services utilization in many countries^1^. Arthritis refers to many diseases that can cause joint pain or stiffness, damage to the structure of a joint, or loss of joint function^2^. The most common types of arthritis are osteoarthritis and rheumatoid arthritis^2^. There are over 100 million people suffering from arthritis in China,which is expected to increase rapidly with the ageing population^3^. It was reported that the overall prevalence of arthritis is 31.4% of Chinese adults aged over 45 years^4^. Arthritis is considered to be the major cause of work disability, leading to a decline in quality of life^5^ and contributes to substantial healthcare costs^2^.

Chinese herbal medicine (CHM) has been attracting global attention over the past 30 years^6,7^. CHM, a key modality of Traditional Chinese medicine (TCM), has been in existence in China for over 2000 years and has been studied for many medical problems, including stroke^8^, mental disorders^9^, respiratory diseases^10^ and arthritis^11^. Specifically, CHM has been shown to have a positive effect on relieving pain and reducing adverse reactions for osteoarthritis (OA) patients^12^. Further, CHM patches combined with Western medicine were suggested to be more effective than Western medicine alone for pain relief of patients with acute gouty arthritis^13^. Furthermore, by adding CHM such as Chuan-niu-xi, Jie-geng, and San-qi into conventional therapy (e.g. methotrexate, leflunomide, calycosin, an O-methylated isoflavone), it led to not only a lower risk of depression but also less incidents of adverse drug reactions for rheumatoid arthritis (RA) patients^14,15^.

Internationally, studies have reported various influencing factors to be associated with the choice of using CHM for arthritis treatment, such as gender, age, education, and health status. Women with arthritis had a higher probability of using CHM^16^. Also, women who used CHM for their arthritis are more likely to have a longer duration of arthritis, have consulted another complementary medicine practitioner, live in a rural area, and undertake more exercise^16,17^. However, influencing factors associated with the choice of using CHM of the patients have not been sufficiently researched. Given the contemporary popularity and significance of CHM use in China and women are more susceptible to arthritis^18,19^, it is vital to identify factors associated with CHM use amongst Chinese women with arthritis to help inform safe, effective and coordinated care. Therefore, in response to this research gap, the aim of this study is to examine the prevalence of CHM usage amongst women with arthritis and the factors associated with the choice of using CHM treatment among those women with arthritis. This research is the first large-scale, nationally representative study of CHM usage in middle-aged and older women with arthritis in China^20^.

## Methods

### Data source and analytical sample

This study is a secondary analysis of the deidentified China Health and Retirement Longitudinal Study (CHARLS) data. CHARLS is conducted by the National School of Development of Peking University since 2011, which is recognized as a nationally representative survey of the middle-aged and elderly population of China^21^. Aimed at investigating multiple factors associated with the rapid aging of the population in China, residents aged 45 or older and their spouses were interviewed, with thorough assessments of social, economic, and health status. The baseline survey involved 17,708 respondents who were recruited through multi-stage probability sampling. They have been followed every 2 years through a face-to-face computer-assisted personal interview (CAPI).

This study analyzed data from the most recent follow-up questionnaire, conducted in 2015. A detailed description of CHARLS was published previously^21^. Women aged 45 years and older who reported having been diagnosed with arthritis were selected for this research. The original CHARLS was approved by the Ethical Review Committee of Peking University, all methods were performed in accordance with relevant guidelines and regulations, and all participants signed informed consent at the time of participation.

### Survey instrument

There were 20,967 subjects participated in the CHARLS 2015 follow-up questionnaire, comprising 10,833 women. The CHARLS questionnaire includes modules such as demographics, health status and functioning, health care and insurance, retirement and pension, and community level information, etc. Since this research is interested in exploring the influencing factors associated with CHM use amongst women with arthritis, the subsample of men was excluded.

### Sociodemographic characteristics

Participants were asked about a number of demographic measures, including age, area of residence (urban or non-urban), marital status (married/de facto or separated/divorced/widowed/never married), and health insurance status (yes or no). Participants were also asked about their lifestyle and health behavior factors, including smoking status and alcohol use.

### Health status

Participants were asked which parts of the body they were currently feeling pain, such as the shoulder, arm, wrist, finger, back, knee, and ankle. Furthermore, participants were asked whether they had difficulty walking, stooping, or reaching above shoulder level. Participants were also asked whether they had fallen down before. Information about their self-rated health status (good, fair, poor) was also asked. Moreover, participants with arthritis were asked to report the time since arthritis diagnosis.

### Health services use

Participants were asked about their visits to a range of medical facilities they had visited in the previous 4 weeks for outpatient treatment, such as Western Medicine hospitals, specialized hospitals and Chinese medicine hospitals. In addition, participants were asked whether they had used self-treatment methods during the past month, including self-purchased over-the-counter Western medicines, vitamins/supplements and consultatations with a massage therapist.

### Outcome measures

Participants were asked to report whether they were currently taking Chinese herbal medicine to treat arthritis, with a yes or no response.

### Statistical analysis

The sociodemographic, health status, and health services use characteristics of CHM users and non-users were compared using chi-squared tests and Fishers Exact tests, where appropriate. Logistic regression modelling, which inititally included all variables that had a bivariate p < 0.2, was conducted using a backward stepwise method, to determine the most parsimonious model for predicting the use of CHM amongst women with arthritis. The model-building process utilized the likelihood ratio test to compare competing models. Statistical significance was set at p < 0.05. All statistical analyses were undertaken using the statistical program Stata (StataCorp LP, College Station, Texas, USA).

## Results

There were 651 (17.2%) out of 3788 women with arthritis who used CHM to treat their arthritis. The average number of years since arthritis diagnosis for women who used CHM and who did not use CHM was 11.8 (SD = 9.5), and 12.7 (SD = 9.6), respectively. Table 1 shows the bivariate comparison of sociodemographic characteristics between women with arthritis who used CHM and those with arthritis who did not use CHM. The only statistically significant difference between CHM users and non-users was observed for the area of residence (p = 0.013), where CHM users were more likely to reside in a rural area.

**Table 1:** Associations between CHM use and demographic characteristics, by Chinese women with arthritis.

Demographic characteristics		CHM use		
		No (n = 3137)	Yes (n = 651)	p-value
		n (%)	n (%)	
Area of residence	Urban	630 (20.5)	103 (16.2)	0.013
	Non-urban	2446 (79.5)	533 (83.8)	
Marital status	Married/de facto	2499 (79.7)	501 (77.0)	0.122
	Separated/divorced/widowed/never married	638 (20.3)	150 (23.0)	
Insurance status	No	293 (9.3)	75 (11.5)	0.087
	Yes	2844 (90.7)	576 (88.5)	
Smoking status	No	2812 (89.6)	586 (90.0)	0.774
	Yes	325 (10.4)	65 (10.0)	
Alcohol status	Non-drinker	2656 (84.8)	545 (83.7)	0.374
	Rarely	215 (6.9)	41 (6.3)	
	Often	262 (8.3)	65 (10.0)	

		Mean (SD)	Mean (SD)	
Age		62.7 (9.6)	62.0 (9.3)	0.103

Table 2 shows the associations between health status and the use of CHM for arthritis. A statistically significant association was observed between self-rated general health status (p < 0.001), and pain of the shoulder (p < 0.001), arm (p < 0.001), wrist (p < 0.001), finger (p < 0.001), back (p < 0.001), waist (p < 0.001), buttocks (p < 0.001), leg (p < 0.001), knee (p < 0.001), ankle (p < 0.001), toe and neck (p < 0.001). In all instances, those women with the respective pain were more likely to use CHM when compared to women without the pain. In addition, women who used CHM for their arthritis were also more likely to have difficulties in running 1 km (p < 0.001), walking 1 km (p = 0.004), stooping/kneeling/crouching (p < 0.001), and reaching or extending arms above shoulder level (p < 0.001) when compared with women who did not use CHM. Further, women who used CHM for their arthritis were more likely to have experienced having a fall (p < 0.001).

**Table 2:** Associations between CHM use and health status, by Chinese women with arthritis.

Health status		CHM use		
		No (n = 3137)	Yes (n = 651)	p-value
		n (%)	n (%)	
General health status	Good	419 (14.1)	47 (7.5)	< 0.001
	Fair	1450 (48.7)	266 (42.7)	
	Poor	1108 (37.2)	310 (49.8)	
Shoulder pain	No	2306 (73.5)	391 (60.1)	< 0.001
	Yes	831 (26.5)	260 (39.9)	
Arm pain	No	2479 (79.0)	430 (66.1)	< 0.001
	Yes	658 (21.0)	221 (33.9)	
Wrist pain	No	2616 (83.4)	457 (70.2)	< 0.001
	Yes	521 (16.6)	194 (29.8)	
Finger pain	No	2562 (81.7)	441 (67.7)	< 0.001
	Yes	575 (18.3)	210 (32.3)	
Back pain	No	2471 (78.8)	429 (65.9)	< 0.001
	Yes	666 (21.2)	222 (34.1)	
Waist pain	No	2083 (66.4)	325 (49.9)	< 0.001
	Yes	1054 (33.6)	326 (50.1)	
Buttocks pain	No	2760 (88.0)	517 (79.4)	< 0.001
	Yes	377 (12.0)	134 (20.6)	
Leg pain	No	2281 (72.7)	368 (56.5)	< 0.001
	Yes	856 (27.3)	283 (43.5)	
Knee pain	No	2173 (69.3)	348 (53.5)	< 0.001
	Yes	964 (30.7)	303 (46.5)	
Ankle pain	No	2630 (83.8)	474 (72.8)	< 0.001
	Yes	507 (16.2)	177 (27.2)	
Toe pain	No	2778 (88.6)	526 (80.8)	< 0.001
	Yes	359 (11.4)	125 (19.2)	
Neck pain	No	2580 (82.2)	462 (71.0)	< 0.001
	Yes	557 (17.8)	189 (29.0)	
Difficulty in walking 1 km	No	1312 (58.9)	262 (51.9)	0.004
	Yes	915 (41.1)	243 (48.1)	
Difficulty in stooping, kneeling, crouching	No	1516 (48.5)	231 (35.5)	< 0.001
	Yes	1609 (51.5)	419 (61.5)	
Difficulty with reaching	No	2570 (82.3)	486 (74.6)	< 0.001
	Yes	554 (17.7)	165 (25.4)	
Fallen down	No	2394 (76.4)	429 (66.0)	< 0.001
	Yes	741 (23.6)	221 (34.0)	

		Mean (SD)	Mean (SD)	
Arthritis time (years since diagnosis)		11.79 (9.5)	12.73 (9.6)	0.059

The associations between women’s use of health services and their use of CHM for arthritis are presented in Table 3. CHM users were more likely to visit a specialized hospital (p = 0.013) and/or a Chinese medicine hospital (p < 0.001). In addition, CHM users were more likely to self-purchase over-the-counter Western medicine medications (p = 0.117) and/or self-purchase vitamins/supplements (p = 0.001), when compared with CHM non-users. Further, women who used CHM were more likely to visit massage therapists (p < 0.001) for their arthritis, compared with those arthritis women who did not use CHM.

**Table 3:** Associations between CHM use and health services use, by Chinese women with arthritis.

Health services choices		CHM use		
		No (n = 3137)	Yes (n = 651)	p-value
		n (%)	n (%)	
Western medicine hospital	No	2889 (92.1)	593 (91.1)	0.392
	Yes	248 (7.9)	58 (8.9)	
Specialized hospital	No	3109 (99.1)	638 (98.0)	0.013
	Yes	28 (0.9)	13 (2.0)	
Chinese medicine hospital	No	3088 (98.4)	625 (96.0)	< 0.001
	Yes	49 (1.6)	26 (4.0)	
Self-purchased over-the-counter western medicine medications	No	1882 (60.0)	369 (56.7)	0.117
	Yes	1255 (40.0)	282 (43.3)	
Self-purchased vitamins/supplements	No	2838 (90.5)	561 (86.2)	0.001
	Yes	299 (9.5)	90 (13.8)	
Massage therapist	No	2958 (94.3)	560 (86.0)	< 0.001
	Yes	179 (5.7)	91 (14.0)	

The statistically significant predictors of CHM use for women with arthritis are presented in Table 4. The Hosmer and Lemeshow goodness-of-fit statistic for this regression model indicated that the model was appropriate (p = 0.660). Women with arthritis who had suffered finger pain (OR = 1.70; 95% CI = 1.39, 2.07) were more likely to use CHM compared to women with no finger pain. Those who visited a massage therapist were more likely to use CHM (OR = 2.06; 95% CI = 1.56, 2.72) than women who did not visit a massage therapist. Women who went to a Traditional Chinese hospital were more likely to use CHM (OR = 2.22; 95% CI = 1.35, 3.64) compared to women who did not go to a Traditional Chinese hospital. Further, experiencing a fall (OR = 1.41; 95% CI = 1.17, 1.70) and/or having difficulty in stooping/kneeling/kneeling/crouching (OR = 1.40; 95% CI = 1.17, 1.68) were associated with higher likelihood of CHM usage.

**Table 4:** Logistic regression identifying the statistically significant predictors of CHM use by Chinese women with arthritis.

Predictors of CHM use		Odds ratio	95% C.I	p-value
Finger pain	No	1.00	–	< 0.001
	Yes	1.70	1.39, 2.07	
Difficulty in stooping, kneeling, crouching	No	1.00		< 0.001
	Yes	1.40	1.17, 1.68	
Fallen down	No	1.00		< 0.001
	Yes	1.41	1.17, 1.70	
Massage therapist	No	1.00		< 0.001
	Yes	2.06	1.56, 2.72	
Traditional Chinese hospital	No	1.00	–	0.002
	Yes	2.22	1.35, 3.64	

## Discussion

In this nationally representative longitudinal survey study of middle-aged and older women with arthritis in China, the prevalence of CHM use was found to be 17.2% (n = 652). This finding is much higher than the results from Australia, indicating that 4% of women aged 62–67 had used CHM for their arthritis^16^. While another study conducted in Taiwan revealed that 83% of women with newly diagnosed RA used CHM^22^. The disparities between the prevalence rates of this research and those previous studies may be explained by several reasons. In the study from Australia, the age group of women was limited to 62–67 years old, which may lead to a conservative estimation of CHM use amongst female participants. As for the relatively higher prevalence of CHM use for women with RA found in Taiwan, the reason may be suggested by systematic reviews showing that CHM has beneficial clinical effects and quality of life improvement on RA, the most common type of inflammatory arthritis^23^. As such, further research is necessary to explore the detailed benefits of specific CHM types of arthritis conditions and symptoms for various age groups of women.

Consistent with previous studies, pain in the fingers was one of the most prevalent complaints reported by patients with arthritis^24^. In addition, results from this research indicated finger pain is one of the predictors that women with arthritis use CHM, which can be explained by the fact that CHM could exhibit strong anti-inflammatory and anti-oxidant activities, contributing to a reduction in inflammation and tissue damage in patients with arthritis^25^. Furthermore, the result from this study is in accordance with an analysis of arthritic adults in the US, suggesting that 21.5% of them used CHM to treat joint pain problems^26^. As the majority of women with arthritis reported pain^27^, further research is warranted to determine tailored treatments (both CHM and conventional) in order to provide better quality arthritis care for women.

This study demonstrates that women with arthritis who used CHM were more likely to experience limitations in physical function, such as stooping/kneeling/crouching, which may be explained by the fact that patients with arthritis suffer from joint damage^28^ and CHM provides potential benefits for physical functional improvement^29^. This result is in accordance with a placebo-controlled double-blind crossover trial indicating that CHM provides an analgesic effect and improved function in comparison to placebo for knee OA patients^30^. Furthermore, women with arthritis were more likely to experience declines in muscle density which could lead to worsening of physical function^28^, while CHM has been shown to have beneficial effects on muscle strength in older women^31^. More research is needed to identify the underlying mechanism of CHM in the physical function of those women with arthritis and related joint symptoms to improve arthritis management.

It is interesting to find from this study that women with arthritis who had fallen down before were more likely to use CHM adds support to the findings of a previous study indicating the fear of falling during daily activities decreased significantly in a Tai chi group, compared to the control group among women with OA^32^. The finding from this study is not surprising given that a wide range of fall incidence reported from people with arthritis, due to disease-related impairments (e.g., decreased muscle strength, reduced functioning, and pain)^33^. Moreover, a cross-sectional study comprising 1273 participants aged 60 years or older in the US observed high risk for falls among older adults who used complementary medicine (CM, including CHM), suggesting CM practitioners to pay particular attention on falls prevention strategies^34^. Nevertheless, more studies are required to examine the finer details of how incidents of falls may influence the choice of CHM use among female arthritis sufferers.

This research shows that CHM usage was positively associated with visiting a massage therapist and/or traditional Chinese hospital for women with arthritis. This result is consistent with the conclusion of a questionnaire-based interview from 250 patients with either RA or OA, indicating most CHM users believed complementary medicine (including massage theray)^35^. Furthermore, women who used CHM were reported to use multiple types of CM for their arthritic problems, such as multi-herb products, massage therapy and acupuncture^15,36,37^. However, the fact that women trying different types of CM could result in potential drug-CM interventions^38^. Further research is required to confirm the effectiveness of CHM on patients suffering from arthritis with regard to differences in gender, and health-related characteristics, in order to provide insights for women with arthritis and healthcare providers by decision making around CHM therapy, conventional treatment, and other healthcare use.

The interpretation of the above findings is limited by the fact that health conditions, health behaviors, and health services use are self-reported by the participants. As a result, findings may be open to recall bias. Moreover, the data analyzed came from a cross-sectional study that could only examine CHM use among women with arthritis, so further information on prescription, dosage and frequency of CHM use were not available in the original data. Arthritis could be diagnosed across the life course^39^, but this research focused on women aged 45 and above, thus limiting the generalizability of findings. Nevertheless, these limitations are countered by the fact that it is the first opportunity to examine middle-aged and older women who used CHM for their arthritis from an established, large, nationally representative sample that could be used to explore the associations between CHM use and women with arthritis.

## Conclusion

Overall, the findings from this study indicate that a considerable proportion of Chinese women with arthritis utilize CHM to help deal with their arthritic symptoms, particularly those with pain and limited physical functioning. These findings reinforce the need for considering integrating CHM use into arthritic care management to improve quality and coordinated arthritis care.

## Data Availability

The datasets generated during and/or analyzed during the current study are available from the corresponding author on reasonable request.
